# Artificial neural network risk prediction of COPD exacerbations using urine biomarkers

**DOI:** 10.1183/23120541.00797-2024

**Published:** 2025-06-02

**Authors:** Ahmed J. Yousuf, Gita Parekh, Malcolm Farrow, Graham Ball, Sara Graziadio, Kevin Wilson, Clare Lendrem, Liesl Carr, Lynne Watson, Sarah Parker, Joanne Finch, Sarah Glover, Vijay Mistry, Kate Porter, Annelyse Duvoix, Linda O'Brien, Sarah Rees, Keir E. Lewis, Paul Davis, Christopher E. Brightling

**Affiliations:** 1 Institute for Lung Health, NIHR BRC Respiratory Medicine, Department of Respiratory Sciences, University of Leicester, Leicester, UK; 2 Mologic LTD (trading as Global Access Diagnostics), Bedford, UK; 3 School of Mathematics, Statistics and Physics, Newcastle University, Newcastle upon Tyne, UK; 4 Medical Technology Research Centre, Anglia Ruskin University, Chelmsford, UK; 5 NIHR Newcastle In Vitro Diagnostics Co-operative, Newcastle University, Newcastle upon Tyne, UK; 6 Prince Philip Hospital, Hywel Dda University Health Board, Llanelli, UK; 7 University of Swansea, Swansea, UK

## Abstract

**Background:**

COPD exacerbations cause considerable morbidity and mortality. We sought to identify a panel of urine biomarkers that can distinguish between stable and exacerbation states and predict risk of future exacerbations.

**Methods:**

A retrospective discovery study was done measuring 35 biomarkers implicated in COPD pathogenesis in paired urine samples from 55 COPD subjects during stable and exacerbation states. A logistic regression model combining the 10 most discriminatory biomarkers in distinguishing between stable and exacerbation states was developed as a near-patient dipstick test with an opto-electronic reader. This biomarker panel was tested in a prospective study of 105 COPD subjects who undertook daily home urine testing over 6 months. The regression model was validated in paired samples from 26 individuals out of 105. An artificial neural network (ANN) using the urine biomarkers from 85 out of 105 subjects was developed and tested as a clinical decision tool to predict risk of an exacerbation.

**Results:**

The 10-biomarker panel (NGAL, TIMP1, CRP, fibrinogen, CC16, fMLP, TIMP2, A1AT, B2M and MMP8) was able to distinguish exacerbation *versus* stable state in the discovery study (ROC with an AUC 0.84, 95% CI 0.76–0.92; p <0.01) and validation study (AUC 0.81, 95% CI 0.70–0.92, p<0.01). The ANN model predicted an exacerbation within a 13-day window frame with an AUC 0.89 (95% CI 0.89–0.90) and identified an exacerbation median (interquartile range) 7 (5–9) days prior to clinical diagnosis.

**Conclusion:**

We identified a panel of biomarkers that can distinguish between stable and exacerbation state, and using an ANN model, it can predict exacerbations before symptoms occur.

## Shareable abstract

**A** **panel of urine biomarkers can be measured with a point-of-care test to identify and predict the likelihood of COPD exacerbations. This suggests that noninvasive and easily measured urine biomarkers are important in COPD.** https://bit.ly/4hBhSgQ

## Introduction

There are over 400 million people with a diagnosis of COPD worldwide [1]. COPD is a heterogeneous and complex disease characterised by persistent symptoms, impaired lung function and episodes of acute worsening of symptoms known as exacerbations. In COPD, exacerbations are the principal driver of poor health status, hospitalisations, mortality and healthcare costs [1–3]. Several clinical features and biomarkers are associated with an increased risk of future exacerbations such as past history of exacerbations, disease severity, blood fibrinogen and blood eosinophils [4–7]. The blood eosinophil count can be used to identify those most likely to respond to maintenance inhaled corticosteroids [8]. At the onset of an exacerbation blood eosinophils and C-reactive protein (CRP) have utility in directing oral corticosteroid and antibiotic therapy, respectively [4, 9–12]. However, these clinical features and blood biomarkers do not provide a reliable risk prediction of the timing of an onset of an exacerbation. It would be attractive to have personalised risk prediction tools that could be used to inform early clinical management decisions, potentially preventing the exacerbation or reducing its severity.

Telemonitoring to predict exacerbations are acceptable to patients but most studies have failed to show benefit in mortality, quality-of-life or cost-effectiveness [13]. Serial assessment of inflammation using frequent blood testing is unlikely to be acceptable to patients, and therefore a less invasive alternative is required. Urine testing presents a simple, safe and reliable alternative and has been used to measure desmosine as a biomarker of emphysema [14]. We hypothesised that a urine biomarker panel can discriminate between stable and exacerbation states in COPD and that an artificial neural network (ANN) utilising serial home testing of this urine biomarker panel can predict the onset of an exacerbation of COPD with acceptable accuracy.

## Material and methods

### Study subjects and design

We undertook a discovery and validation study of urine biomarkers as predictors of COPD exacerbations. In both studies, an exacerbation was defined as an acute event characterised by a worsening of the patient's respiratory symptoms beyond normal day-to-day variations leading to the administration of systemic corticosteroids and/or antibiotics [1]. For the inclusion and exclusion criteria, please see the supplementary material.

The discovery study was *post hoc*. Paired urine samples obtained at stable and exacerbation visits were available from 55 COPD subjects that had participated in a previously reported single centre study undertaken in Leicester, UK [11, 12]. Subjects were assessed at baseline, 3, 6, 9 and 12 months and at exacerbation. The validation study was prospective and performed in two centres, Llanelli and Leicester, UK from July 2017 to February 2018. These subjects were enrolled into the 6-month observational study for scheduled stable 6-weekly visits and unscheduled exacerbation visits. The studies were approved by the local ethics committee and subjects gave written informed consent (ethical approval 08/H0406/189).

In both studies demographic data including age, medical history, medications, smoking history and previous exacerbation history were recorded. Spirometry, respiratory symptoms and health-related quality of life using the St George's Respiratory Questionnaire were recorded at each time point.

In the discovery study urine samples were collected at scheduled stable and unscheduled exacerbation visits and were stored at −80°C until analysis. Urine concentrations of 35 biomarkers implicated in the pathogenesis of COPD [4, 5, 7, 9, 10, 12, 14, 15–19] using ELISA or lateral flow assays were analysed according to the manufacturer's instructions (supplementary table S1). All measurements were done in duplicate by a blinded observer.

The urine biomarkers in the discovery study that were discriminatory between stable and exacerbation visits were optimised as lateral flow assays that were applied to two multiplex urine dipsticks (10 assays) read by a commercially available opto-electronic reader. The technical development of the urine biomarker assay Headstart is described in the supplementary material with a diagrammatic representation of the design (supplementary figure S1).

A patient usability questionnaire was completed at the end of the study to evaluate key parameters such as usability/ease-of-use, frequency of testing, data transfer, safety, practicalities and recommendations/improvements (supplementary table S2). Compliance to using the device was also assessed throughout the validation study.

In the validation study COPD subjects undertook daily urine tests, read in 10 min with data transferred by Bluetooth to a mobile phone and uploaded with daily diary in addition to their study visits. This study allowed us to test the discrimination of the original biomarker panel at baseline *versus* exacerbations.

The daily measures undertaken in the validation study allowed us to refine the biomarker panel from 10 to 5 biomarkers, to develop a predictive ANN model assessing “time to exacerbation” and to develop a risk score to estimate the probability of a future exacerbation occurring.

Thus, the 10-plex algorithm was used to evaluate the robustness of the regression model across two different studies. This regression model was developed to differentiate between stable and exacerbation states only. The 5-plex ANN algorithm was developed using data from the prospective study using daily samples to predict an impending exacerbation.

### Analysis

#### Statistical analysis and urine biomarker panel predictive neural network model

Statistical analyses were carried out with PRISM (version 8.4.3) and SPSS (version 25). In the discovery phase the differences in paired urine concentrations of 35 biomarkers between stable state and exacerbation were evaluated by t-test and receiver operating characteristics (ROC) curves. Our predefined selection criteria for each biomarker were an area under the curve (AUC) ≥0.59 or ≤0.41 and p<0.05. A backward stepwise regression was used, which removed the least significant variable (*i.e.*, p>0.05) at each step. In the validation phase, to test the diagnostic accuracy of the logistic regression model, ROC analysis was used to quantify the biomarker performance by means of sensitivity, specificity, AUC with corresponding 95% confidence intervals.

The biomarkers that were discriminatory between stable and exacerbation visit were tested prospectively in the ANN model. The ANN model followed our previously reported approach in biomarker evaluation for breast cancer [20–22]. The ANN model was applied to filtered values, which were produced by applying a specially developed Kalman filter to the data from each patient. The filter was developed by first fitting a non-homogeneous dynamic linear model (DLM) to the data from the validation study using the two multiplex lateral flow assays. Further details of the Kalman filter and DLM are given in the supplementary material. The logarithms of the concentrations were used to stabilise the variance and to make the effects of sample dilution additive. As well as the usual observational error term, the DLM allowed for individual patient baselines and for a slow drift in this over time, and for step changes associated with changes of test device batch. A separate univariate model was fitted to data from each biomarker. The model was fitted by Bayesian inference with vague prior distributions given to the four variance parameters in the DLM and to an autoregressive coefficient associated with short-term fluctuations. Assessment of central values for the prior distributions for the variances was assisted by the fact that the logarithms of concentrations were used. This meant that prior judgements about the magnitude of local, that is day-to-day, variations could be expressed in terms of proportions of the local level of the untransformed data, such as “plus or minus 10%”, rather than in terms of absolute values. A Markov chain Monte Carlo (MCMC) method was used to fit the model. As a result, cases with missing observations were handled easily since, at each iteration of the MCMC algorithm, a random sample is taken from the posterior distribution of all of the unknowns, including the missing observations as well as the unknown parameters. It was assumed that missingness of an observation was not informative. The filter algorithm was then developed, based on the results of the model fitting. While the model fitting used the whole of the available data series, the value supplied by the filter for the ANN comprised filtering up to a particular day providing filtered values for that particular day. Thus, filtered values were provided for each individual case for each day of the study.

Data used for the development of the algorithm consisted of days leading up to an exacerbation and randomised stable days. The recovery period (6 weeks after a confirmed exacerbation) was excluded due to the possibility that resolution of symptoms and inflammation would take 6 weeks. These data were used as an additional blinded dataset (production set). Included in the production set was also data from patients who experienced no exacerbations during the study.

For each day of testing for each individual, a time to exacerbation was assigned based on clinician diagnosis. A window of 13 days was used to generate a rolling profile for each patient for each biomarker, leading up to the day of exacerbation. 13 days was selected so as to provide a lead in time to an exacerbation allowing prediction of an exacerbation up to 13 days before it occurred. Smaller windows were also explored but these showed poorer performance. For each day in the profile, the value on a given day, the differential from the previous day and the integral (sum of values) up to that day were taken. Thus, for each marker for a given window, 42 values were determined. This window was then rolled through the data incrementing one day at a time. This was then repeated for each biomarker. Thus, a database was generated for each individual in the population, comprising the individual's temporal biomarker profile and a time to exacerbation. Individuals with no exacerbations were set with a standard time to exacerbation of 200 days. 200 days was selected as this was greater than the longest observational period for any individual on the trial and thus was set to represent a time-to-exacerbation longer than this period.

These data were mined using a stepwise ANN selecting the best combination of temporal-biomarkers up to 20 steps [20]. The dataset was randomly split into three sets, training, validation and test using a 60:20:20 split respectively. Multiple runs of this stepwise process were completed using different Monte Carlo cross-validation splits in order to determine the variability/stability of the biomarker panels for the data across different randomised splits. Biomarker panels were selected based on their performance on the unseen test set data. The output from the ANN analysis was a risk score, which represented “time to exacerbation”. On completion of the parameter selection phase, the model was further tuned varying the number of hidden nodes to further optimise the performance on the unseen test set data. This model was further evaluated and converted into a mathematical formula for utilisation.

For training, validation and test set data, Pearson correlation for actual time-to-exacerbation against predicted time-to-exacerbation was used to confirm performance of the ANN model. ROC analysis was used to assess the clinical performance of the biomarkers at different time cut-off points (exacerbation window period before a clinician diagnosis). Using the training, validation and test sets and the unseen blinded production set, decision rules were applied, categorising the risk score into a red/amber/green outcome. If the risk score was above or below the cut-off, it was attributed as a “green” or “amber” outcome, respectively. If there were 6 out of 7 days consecutive “amber” outcomes, then the result would be attributed as a “red” outcome. A true positive (TP) was obtained if a “red” result fell into the exacerbation window, a false negative (FN) if there was an absence of a “red” result within the exacerbation window, a false positive (FP) if a “red” result was obtained outside the window and a true negative (TN) if a “green” outcome was obtained in the stable periods. Positive predictive value (PPV) and negative predictive value (NPV) were determined (PPV=100×TP/(TP+FP) and NPV=100×TN/(FN+TN)).

## Results

In the retrospective “discovery” dataset, paired urine samples obtained at stable visits and exacerbations were available for analysis in 55 of 145 recruited COPD subjects (supplementary figure S2a). In the prospective “validation” study, 105 subjects were recruited of which 26 subjects had 33 exacerbations confirmed by study-physician (supplementary figure S2b). These 26 subjects were included because they had paired stable and exacerbation samples. The baseline clinical characteristics of these subjects were similar and are shown in table 1.

**TABLE 1:** Baseline characteristics of patients

	Discovery	Validation	ANN
**Patients, n**	55	26	85
**Age years, median (range)**	69.0 (43–84)	70.0 (50–80)	72 (50–84)
**Male, n (%)**	37.0 (67)	18.0 (69)	61.0 (72.0)
**White ethnicity, %**	100	100	100
**Current smoker, n (%)**	9.0 (16.0)	6.0 (23.0)	18 (21.0)
**Ex-smoker, n (%)**	46.0 (84.0)	21.0 (77.0)	67.0 (79.0)
**Pack-years, median (range)**	49.0 (10–153)	52.0 (11–120)	47.0 (11–156)
**BMI kg**·**m^−2^, mean±****se**	25.6±0.70	28.5±1.16	27.0±0.60
**Post BD FEV_1_ % pred, mean±** **se**	50.6±2.5	46.65±3.87	55.80±2.35
**FEV_1_/FVC ratio %, mean±** **se**	50.0±1.0	46.5±2.6	50.6±1.48
**MRC score, mean±** **se**	3.0±0.1	3.0±0.2	3.0±0.13
**SGRQ score, mean±** **se**	48.1±2.7	58.61±3.8	48.0±2.47
**CAT score, mean±** **se**	Not recorded	24.0±1.2	21.0±0.92
**Sputum eosinophils %, median (IQR)**	1.2 (0.25–4.20)	2.0 (0.4–7.1)	1.0 (0.25–3.5)
**Sputum neutrophils %, median (IQR)**	69.7 (45.38–90.56)	69.7 (39.0–80.4)	72.75 (42.75–89.38)
**GOLD stage, n (%)**			
** **I	0	1 (3.8)	11 (12.9)
** **II	27 (49.0)	9 (34.6)	38 (44.7)
** **III	20 (36.0)	11 (42.3)	24 (28.2)
** **IV	7 (13.0)	5 (19.2)	12 (14.1)

ANN: artificial neural network; BMI: body mass index; BD: bronchodilator; FEV_1_: forced expiratory volume in 1 s; FVC: forced vital capacity; MRC: Medical Research Council score; SGRQ: St George's Respiratory Questionnaire; CAT: COPD Assessment Test; GOLD: Global Initiative for Chronic Obstructive Lung Disease.

From the 35-biomarker panel (supplementary table S2), 10 biomarkers met our predefined selection criteria: fibrinogen, formyl-methionine-leucine-phenylalanine (fMLP), tissue inhibitor of metalloproteinase 2 (TIMP2), CRP, β_2_-microglobulin (B2M), neutrophil gelatinase-associated lipocalin (NGAL), α_1_-antitrypsin (A1AT), club cell protein 16 (CC16), tissue inhibitor of metalloproteinase 1 (TIMP1) and retinal binding protein 4 (RBP4) (supplementary table S3).

The performance characteristics of ELISAs and lateral flow assays (Headstart) are described in supplementary tables S4–S7. The tests also met acceptability and usability criteria, as shown in supplementary figure S3. Daily testing with the test devices collectively had >85% compliance, while the usability success rate was 93% (supplementary figure S4). The mean compliance for the e-diary was 76.5%. From the 26 314 test results, 87.7% (23 077/26 314) tests were done, and 93% (21 461/23 077) were done correctly (*i.e.*, there was a positive control line).

**FIGURE 1:**
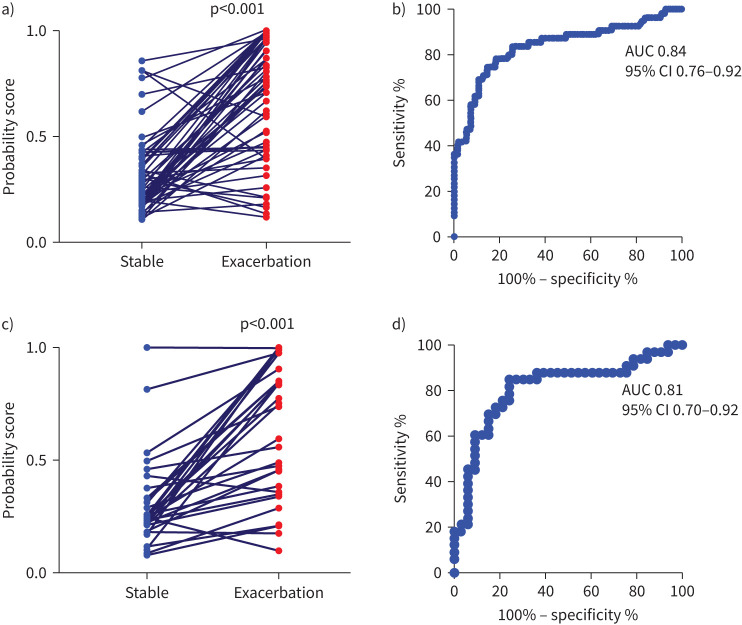
Probability of exacerbation using 10-biomarker panel in a,b) discovery cohort and c,d) validation cohort. AUC: area under the curve.

With the 10 biomarkers a probability score was derived for each subject using a logistic regression model. In the discovery dataset (n=55) the probability score was higher in the exacerbation *versus* stable samples, 0.76 (95% CI 0.59–0.86) *versus* 0.25 (95% CI 0.21–0.33) (figure 1a) with ROC AUC of 0.84 (95% CI 0.76–0.92; p<0.001) (figure 1b). In the validation dataset (n=33) the probability score was higher in the exacerbation *versus* stable samples 0.75 (95% CI 0.46–0.85) *versus* 0.24 (95% CI 0.21–0.30) (figure 1c) with a ROC AUC 0.81 (95% confidence interval 0.70–0.92, p<0.001) (figure 1d). Using a probability score cut-off of 0.39, the optimal sensitivity and specificity values for the discovery cohort was 83.6% and 74.5% and for the validation cohort was 72.7% and 78.8%, respectively. The PPV and NPV, using a prevalence of 50%, were 76.7% and 82.0% for the discovery cohort and 77.4% and 74.3% for the validation cohort.

### ANN algorithm results

Of the 105 subjects in the prospective study 85 were included in the ANN model as nine withdrew and 11 had missing data (supplementary figure S2b). The clinical characteristics of the subjects included in the ANN are shown in table 1.

**FIGURE 2:**
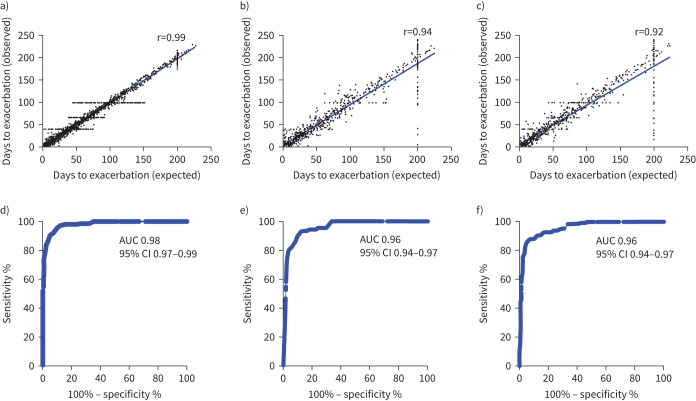
Actual time to exacerbation against predicted time to exacerbation derived from the artificial neural network (ANN) model utilising the a) training, b) validation and c) test datasets. Receiver operating characteristics (ROC) curves to distinguish exacerbations *versus* stable in 13-day windows for d) training, e) validation and f) test datasets. AUC: area under the curve.

**FIGURE 3:**
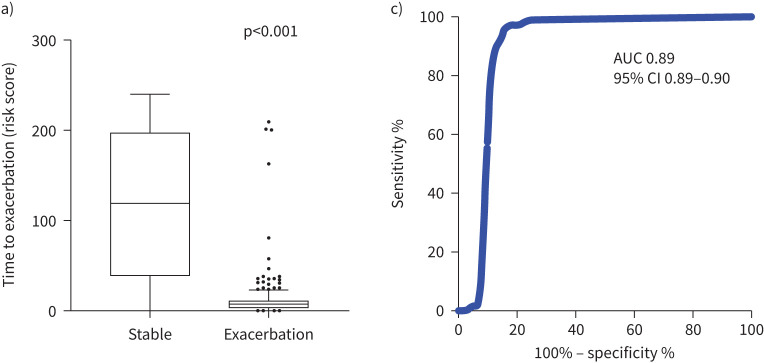
For entire dataset including unseen “production” data: a) scatter plot of the predictive time to exacerbation (risk score) for stable *versus* exacerbation and b) receiver operating characteristics (ROC) curve for stable *versus* exacerbation (13-day window). AUC: area under the curve.

From the multiple stepwise runs of the algorithm using Monte Carlo cross-validation, an optimised panel of five markers derived from the 10 biomarkers was defined as the “Headstart panel”, comprising NGAL, TIMP1, CRP, fibrinogen and CC16. This panel was the most stable and had the best performance for unseen test cases in the ANN model. Using the dataset, the r^2^ values for actual time to exacerbation against predicted time to exacerbation for the training, validation and test data sets were 0.99, 0.94 and 0.92 (figure 2a–c). Multiple exacerbation windows were evaluated, to determine the optimal sensitivity and specificity within a clinically meaningful time frame as shown in supplementary figure S5. From these data a 13-day window was selected (supplementary figure S5). We examined the association between a result from the Headstart Test system and a clinician-confirmed exacerbation (13-day window) through logistic regression with a dependent variable of a clinician-confirmed exacerbation and independent variable Headstart risk score. With this exacerbation 13-day window the ANN model ROC AUC for detecting an exacerbation using the training, validation and test datasets were 0.98 (95% CI 0.97–0.99), 0.96 (95% CI 0.94–0.97) and 0.96 (95% CI 0.94–0.97) respectively (figure 2d–f). Utilising all the data from the study including the blinded “production” set (n=9406 days), the Headstart panel predicted an exacerbation within a 13-day window frame with an AUC of 0.89 (95% CI 0.89–09) with a sensitivity and specificity of 95% and 85%, respectively, using a cut-off of 21.19 (figure 3a, b).

**FIGURE 4:**
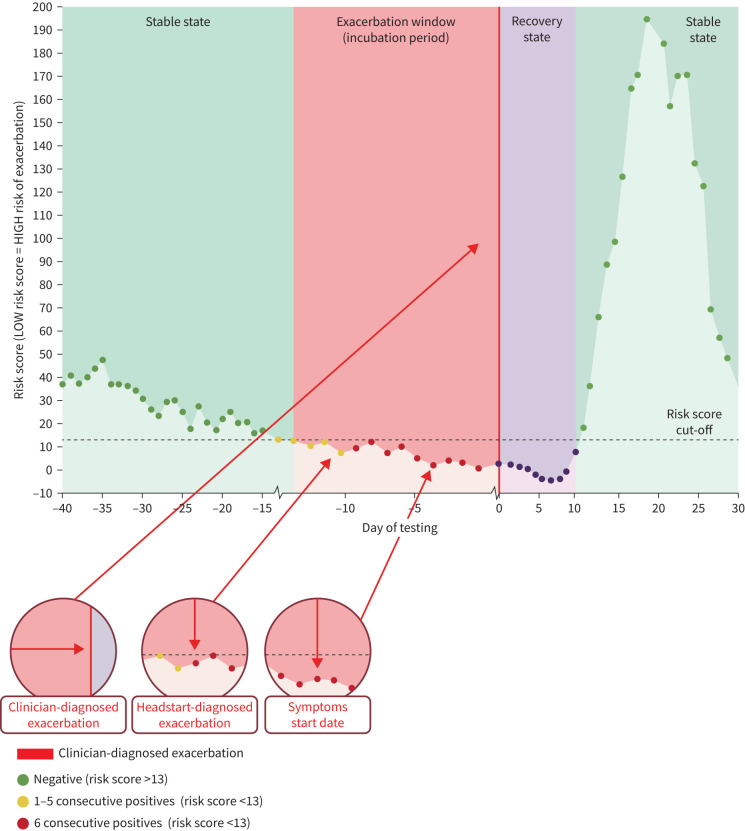
An example of a Headstart Risk Profile for a patient for their first exacerbation using the artificial neural network (ANN) algorithm. Based on selected decision rules, a green negative result is obtained if >13.4 and amber if <13.4 and 6/7 ambers=a red result. For this patient, Headstart was able to diagnose an exacerbation 8 days prior to a clinical diagnosed exacerbation and 5 days prior to symptoms.

A “red” outcome was attributed when there were at least 6/7 consecutive days of “amber” outcomes in a 13-day window. An example patient profile is displayed in figure 4. The sensitivity (out of 43 events) and specificity (out of 8806 stable days) of the ANN model-derived decision rules to determine an exacerbation *versus* stable state was 95% and 91% respectively. Using the described decision rules, a series of consecutive red outcomes is defined as one predicted exacerbation event rather than multiple events; therefore when referring to FPs, a series of “red” outcomes is defined as one FP. Consequently, 766 FP days equated to 38 predicted FP events (exacerbations), and the resulting PPV and NPV using a prevalence of 0.5% were 51.90% and 99.98% respectively. The median (IQR) time that Headstart identified an emerging exacerbation, prior to clinical diagnosis, was 7 (5–9) days. Following an exacerbation, 88% of patients had returned to a stable “non-red” outcome by day 14.

## Discussion

This is the first report of a panel of urinary biomarkers that can discriminate between stable and exacerbation state in a discovery study, which were replicated in a validation study. To detect these biomarkers, we developed a reliable home monitoring device using lateral flow testing of daily urine samples with remote connectivity that was acceptable to patients with COPD and allowed serial measurements of the urinary inflammatory profile. An ANN model that used the serial urine biomarker panel data was able to predict an exacerbation 7 days before the clinical diagnosis.

The definition of a COPD exacerbation relies on self-reported worsening of symptoms [1]. Blood tests, particularly in a clinic or inpatient setting, help to phenotype the heterogeneity of the biology and severity of a COPD exacerbation [4, 5, 7, 9–11, 18, 19, 23–25]. Currently, assessments in the home typically focus on telemonitoring approaches [13], and although personalised early warning systems have shown some promise, they do not currently include personalised assessment of the patient's inflammatory profile [26, 27]. To date biomarkers are not routinely used to support the diagnosis of COPD nor are serial biomarkers used to predict the onset of an exacerbation. Serial blood testing, particularly in the community or in the home, is challenging with urine testing an attractive alternative noninvasive test. Here we report for the first time that a panel of urine biomarkers in patients with COPD can distinguish between samples obtained during stable state *versus* an exacerbation with good accuracy. The biomarker panel was identified in a retrospective discovery study and replicated in a prospective multicentre validation study.

To address the challenge of a personalised early prediction tool we developed a near-patient home monitoring system Headstart to assess the urinary biomarker profile over time. Headstart was technically reliable, patients found the test acceptable and compliance was very high. One of the strengths of our approach was that this enabled frequently measured high-dimensional data amenable to artificial intelligence and machine learning approaches to develop a personalised risk tool. An important step to develop a usable home-testing device was to minimise the panel of biomarkers. The smaller panel derived from the comparisons of stable and exacerbation visits was a pragmatic approach to reduce the size of the panel, and thus whether a larger or alternative panel might have had greater discrimination and prediction is unknown. Our ANN model was able to predict the onset of an exacerbation event from a biological perspective a week earlier than the clinical presentation. In our study, we recruited patients with a prior exacerbation history. However, even with a population enriched for a risk of future exacerbations, these events were rare compared to the background of stable disease days. When we applied our ANN model to a clinical decision tool that included the need for successive days with raised biomarkers, we found it had excellent NPV suggesting that it was very unlikely to miss a clinically important event but had poor PPV identifying approximately double the number of events than those that presented clinically. The PPV is likely to improve in a patient group with a higher prevalence of exacerbations suggesting the clinical utility is probably greatest in those with more frequent exacerbations. Whether urine biomarker testing and the application of the predictive model can inform clinical decisions that improve patient outcomes requires further safety, feasibility, effectiveness and cost-effectiveness studies.

Our study has a number of limitations. The sample sizes in both studies were small. We had paired urine samples for only 55 patients in the discovery study and only 26 patients (33 exacerbations) in the validation cohort. We are confident that the self-managed COPD exacerbations were likely due to COPD rather than alternative causes of the increased symptoms such as heart failure, pneumothorax or pulmonary embolus because additional specific therapies for these causes were not needed, but whether the biomarker panel is specific for COPD exacerbations requires further validation. We did not have a “gold standard” biomarker to compare our results against. The absence of a “gold standard” biomarker for identifying COPD exacerbations impedes the understanding of the real usefulness of urine profile or any other biomarker in distinguishing between stable and exacerbation states in COPD. We selected biomarkers that had biological plausibility and could be robustly analysed in urine and we did targeted assays. Therefore, other biomarkers might exist that are more discriminatory that were not included. The five selected biomarkers were the acute phase proteins (CRP and fibrinogen), a neutrophil product contributing to airway inflammation and remodelling (NGAL), a protease inhibitor (TIMP-1) and a lung-specific marker (CC16). Importantly, each component of the 5-plex biomarker panel have been implicated previously in COPD and COPD exacerbations [15–18], although this is the first study to identify their utility in concert. The biomarker identifies the onset of an exacerbation event but does not identify the aetiology. This would require an additional test such as assessment of eosinophilic inflammation or detection of a pathogen such as by qPCR that could then direct anti-inflammatory or antimicrobial therapy. Some biomarkers such as desmosine, ChI3LI, IL6, SLPI and RNASE3, which have been studied in COPD, were not included in the longitudinal study as they did not meet the performance criteria with paired samples. Urine creatinine levels change significantly at exacerbation from the levels observed in the stable state, but it was not included in the final assay due to the influence of sex, age, body mass and renal function on its levels in urine. Due to the small sample size, we did not explore the potential impact of covariates upon the multiplex panel such as age, sex, ethnicity and body mass index. However, the algorithm calculates changes from personalised baselines for each patient, and therefore these differences are unlikely to affect the individualised predictive model. The test is most reliable in patients with moderate-to-severe COPD with frequent exacerbations, as the ANN model requires sufficient exacerbations to learn the individual biomarker profile and predict future events and thus is unlikely to have utility in those people with infrequent exacerbations. The findings from the ANN model are undertaken in a small study with inherent risks of possibly overfitting the model. The ANN model needs to be further validated to determine generalisability and potential for clinical utility. Usability and acceptability will also need further validation, as we had information available only on a subgroup of participants. Additionally, the question of whether other noninvasive biomarkers, such as those in breathomics, could have similar utility also deserves further study [28].

In conclusion, we have shown that practically detectable changes in urine concentration of inflammatory biomarkers support their use as biomarkers of the airway inflammatory response in COPD exacerbations. Our findings suggested that the identified urine biomarkers are promising in discriminating COPD exacerbations from stable COPD and can be measured at home with a lateral flow reader and mobile technology and so could be used at scale. They can identify biological changes as early as 7 days prior to a clinical diagnosis of a COPD exacerbation. These findings need to be validated in a much larger, multicentre, longitudinal, prospective interventional study, and cost-effectiveness needs to be demonstrated before possible implementation in the clinic.

## Supplementary material

10.1183/23120541.00797-2024.Supp1 **Please note:** supplementary material is not edited by the Editorial Office, and is uploaded as it has been supplied by the author.
Supplementary material 00797-2024.SUPPLEMENT
