# Supplementary material for: Artificial neural network risk prediction of COPD exacerbations using urine biomarkers
Source: ERJ Open Res. 2025 Jun 2;11(3):00797-2024. doi: 10.1183/23120541.00797-2024 (PMC12134921; doi:10.1183/23120541.00797-2024)

Supplementary appendix

Artificial neural network risk prediction of chronic obstructive pulmonary disease (COPD) exacerbations using urine biomarkers

Ahmed J Yousuf[1], Gita Parekh[2], Malcolm Farrow[3], Graham Ball[4], Sara Graziadio[5], Kevin Wilson[3], Clare Lendrem[5], Liesl Carr[1], Lynne Watson[2], Sarah Parker[1], Sarah Glover[1], Vijay Mistry[1], Annelyse Duvoix[2], Linda O'Brien[6], Sarah Rees[6], Keir E. Lewis[6,7], Paul Davis[2], Christopher E. Brightling, FMedSci[1]

[1] Institute for Lung Health, NIHR BRC Respiratory Medicine, Department of Respiratory Sciences, University of Leicester, Leicester, UK;

[2] Mologic LTD (trading as Global Access Diagnostics), Bedford, UK

[3] School of Mathematics, Statistics and Physics, Newcastle University, UK

[4] Medical Technology Research Centre, Anglia Ruskin University, Chelmsford UK

[5] NIHR Newcastle In Vitro Diagnostics Co-operative, Newcastle University, UK

[6] Prince Philip Hospital, Hywel Dda University Health Board, Wales, UK.

[7] University of Swansea, Wales, UK.

Corresponding author: Christopher Brightling PhD FMedSci

Institute for Lung Health, NIHR BRC Respiratory Medicine, Department of Respiratory Sciences, University of Leicester, Leicester, UK

University of Leicester

Leicester, UK;

+44 116 250 2704 ceb17@le·ac·uk

Inclusion and exclusion criteria

Discovery study

- **Inclusion criteria**
  1. Age > 40 years
  2. Post 400mcg salbutamol bronchodilator FEV_1/FVC ratio <0.7
  3. ≥ 1 exacerbation in the previous year requiring corticosteroids and/or antibiotic therapy, including subjects that required hospitalisation for an exacerbation of COPD
- Exclusion criteria
  1. Inability to produce sputum following the induced sputum procedure
  2. A current or previous history of asthma
  3. Currently active pulmonary tuberculosis
  4. Any other clinically relevant lung disease other than COPD
  5. Pregnancy or lactation

Validation study

- **Inclusion criteria**

1. Aged 40 years or over
2. Diagnosis of COPD (GOLD I-IV)
3. Current or ex-smokers with a smoking history of at least 10 pack-year
4. Be willing and able to comply with study procedures and be available for study visits
5. Be a frequent exacerbator or have at least 1 exacerbation per 6 months
6. Be able to give valid written consent

- **Exclusion criteria**

1. Inability to give informed consent

2. Known respiratory disorders other than COPD (asthma, lung cancer, sarcoidosis and other ILDs, tuberculosis, lung fibrosis, cystic fibrosis, and non-COPD related bronchiectasis).

3. Known history of significant systemic and organ-related inflammatory disease, other than COPD (e.g. rheumatoid arthritis and Lupus, kidney, liver, endocrine disorders).

4. Known to be severely alpha-1-antitrypsin deficient (PI SZ or ZZ).

5. Having undergone lung surgery (e.g. lung reduction, lung transplant).

6. Have cancer or have had cancer in the 5 years prior to study entry.

7. Any serious, uncontrolled disease (including serious psychological disorders) likely to interfere with the study or impact on subject safety.

8. Have, in the opinion of the investigator, evidence of alcohol, drug or solvent abuse.

10. Is on long term oral corticosteroids (long term is considered use for more than 3 consecutive months).

11. Unable to walk.

12. Serious co-morbid condition which would interfere with urine marker analysis.

13. Pregnancy

14. Participation in an interventional clinical study within 3 months of Visit 1 or participation in a study using an investigational medicinal product in the previous 4 months.

15. Upon questioning the patient has known HIV infection or positive hepatitis B or C.

Table S1. Biomarker panel (n=35) and assay format [24 commercial (C) and 16 developed in-house (IH)]

	Biomarker	Assay	Analyte (full description)
1	IL-6	ELISA (C)	Interleukin-6
2	fMLP	ELISA (IH) & Lateral flow (IH)	N-Formylmethionine-leucyl-phenylalanine
3	Il-1β	ELISA (C)	Interleukin-1β
4	IL-8	ELISA (C)	Interleukin- 8
5	Siglec 8	ELISA (IH)	Siglec 8
6	CHI3L1	ELISA (C)	Chitinase 3 like protein
7	Active MMP	Lateral flow	Active protease (Composite MMP 2,8,9,12,13)
8	Active MMP	Plate assay (substrate assay) (C)	Active protease (Composite MMP 2,8,9,12,13,7)
9	Active HNE	Plate assay (substrate assay) (C)	Active Human Neutrophil Elastase
10	MMP-8	ELISA (C)	Total Matrix Metalloproteinase -8
11	MMP-9	ELISA (C)	Total Matrix Metalloproteinase -9
12	HNE	ELISA (IH)	Human Neutrophil Elastase
13	NGAL	ELISA (C)	Neutrophil gelatinase-associated lipocalin
14	Calprotectin	ELISA (C)	Calprotectin
15	MPO	ELISA (C)	Myeloperoxidase
16	A1AT	ELISA (IH) & Lateral flow (IH)	Alpha-1 antitrypsin
17	TIMP-1	ELISA (C) & Lateral flow (IH)	Tissue inhibitor of metalloproteinase-1
18	TIMP-2	ELISA (C) & Lateral flow (IH)	Tissue inhibitor of metalloproteinase-2
19	SLPI	ELISA (IH)	Secretory leukocyte protease inhibitor
20	Cystatin C	ELISA (C)	Cystatin C
21	Ac-PGP	ELISA (IH)	N-acetyl Proline-Glycine-Proline
22	Desmosine	ELISA (IH) & Lateral flow (IH)	Desmosine
23	LEF	ELISA (IH)	Large Elastin Fragments
24	Desmosine	ELISA (IH)	Desmosine fragments
25	CC16	ELISA (C)	Club cell- 16
26	CRP	ELISA (C) & Lateral flow (IH)	C reactive protein
27	Periostin	ELISA (C)	Periostin
28	Creatinine	Plate assay (C)	Creatinine
29	B2M	ELISA (C)	beta 2 Microglobulin
30	RBP-4	ELISA (C)	Retinol binding protein-4
31	HSA	ELISA (C)	Human Serum Albumin
32	Fibrinogen	ELISA (C)	Fibrinogen
33	sRAGE	ELISA (IH)	Soluble receptor for advanced glycation end products
34	RNASE-3	ELISA (C)	Eosinophil cationic protein
35	MBP	ELISA (C)	Major Basic protein

Headstart® technical development

Headstart® measuring 10 biomarkers in total was split into 2 lateral flow tests (multiplex A and multiplex B). The design is illustrated in **Figure S1**.

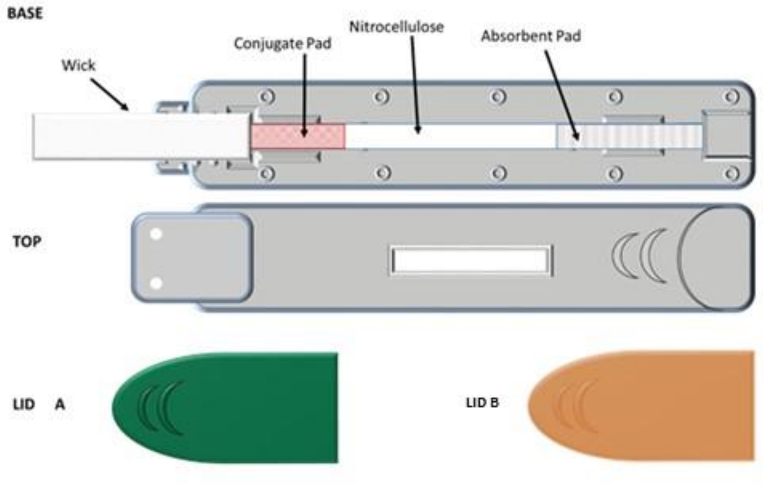

Figure S1. Diagrammatic representation of assembly process for test device version 2 with a green lid for A and an orange lid for B. Manual addition of strip and wick but machine closed to ensure equal distribution of pressure.

Headstart® Multiplex A

Multiplex A consisted of 5 biomarkers, A1AT, TIMP2, NGAL, Fibrinogen and CRP, all 5 were sandwich assays. Two of the assays (A1AT and NGAL) were refined as there was a strong hook effect with high levels of these biomarkers. As a consequence, there was an additional pre-absorbent line for A1AT and free antibody added in the system for the NGAL assay to give just the right assay range.

For the nitrocellulose membrane (NC) all test lines were prepared at 1mg/ml in PBS 1% sucrose. Anti-A1AT pre-absorbent line was plotted at 3mm, anti-A1AT was plotted at 7mm, anti-TIMP2 was plotted at 10mm, anti-NGAL was plotted at 13mm, anti-Fibrinogen antibody

at 16mm, anti-CRP was plotted at 19mm, and control line was plotted at 2mg/ml with 10% green food dye at 22mm. All reagents were plotted onto nitrocellulose at a deposition rate of 0.05µl/mm. Materials were dried in the Hedinair drier at 60ºC at 10mm speed and heat sealed in a foil pouch with 5 x 1g desiccant and cured at 37°C incubator for 18h.

Anti-A1AT antibody (15µg/ml), anti-TIMP2 antibody (15µg/ml), anti-NGAL antibody (15µg/ml), anti-fibrinogen antibody (15µg/ml) and anti-CRP antibody (15µg) were all conjugated to 40nm OD5 gold and blocked with BSA. Conjugates were added together to make up the final OD of 5 for A1AT, TIMP2, NGAL, Fibrinogen, OD10 for CRP and OD2 for control gold conjugate in gold drying buffer + 15µg/ml final of free anti-NGAL antibody. The final conjugate mix was sprayed onto a glass fibre conjugate pad at a deposition rate of 0.8µl/ml. Materials were dried in the Hedinair drier at 60ºC at 5mm speed and stored in a foil pouch with 5 x 1g desiccant.

Headstart® Multiplex B

Multiplex B consisted of 5 biomarkers, RBP4, CC16, B2M, TIMP1 and fMLP. With the exception of TIMP1, all were inhibition assays.

For the NC all capture lines were prepared in PBS 1% sucrose. RBP4 was plotted at 1.5mg/ml at 7mm, CC16 was plotted at 0.05mg/ml at 10mm, B2M was plotted at 0.5mg/ml at 13mm, anti-TIMP1 antibody at 1mg/ml at 16mm, Ovalbumin-fMLP was plotted at 1mg/ml 19mm and control line was plotted at 2mg/ml with 10% orange food dye at 22mm. All reagents were plotted onto nitrocellulose at a deposition rate of 0.05µl/mm. Materials were dried in the Hedinair drier at 60ºC at 10mm speed and heat sealed in a foil pouch with 5 x 1g desiccant and cured at 37°C incubator for 18h.

Anti-RBP4 antibody (15µg/ml), anti-CC16 antibody (15µg/ml), anti-B2M antibody (15µg/ml), anti-TIMP1 antibody (15µg/ml) and anti-fMLP antibody (15µg/ml) were all conjugated to 40nm OD5 gold and blocked with BSA. Conjugates were added together to make up the final OD of 5 for TIMP1 and B2M, OD10 for RBP4, CC16 and fMLP and OD2 for control gold conjugate in gold drying buffer. The final conjugate mix was sprayed onto glass fibre conjugate pad at a deposition rate of 0.8µl/ml. Materials were dried in the Hedinair drier at 60ºC at 5mm speed and stored in a foil pouch with 5 x 1g desiccant.

For both Multiplex A and B lamination onto 80mm backing card, the base of the NC was placed at 25mm height, the base of the conjugate pad was lined up flush with the base of the backing card. The absorbent pad lines up flush to the top of the backing card forming a 7mm overlap with the top of the NC. The strips were cut into 5mm wide strips. Each strip was placed into the cassette housing with a wick and sealed shut using a specific device closing machine and a cap was placed (green for device ‘a’ and orange for device ‘b’) before sealing in individual foil pouches with 1g desiccant.

**Table S2.** Headstart® patient acceptability/ usability questionnaire
1. How often would you be happy to use the test?<br>2. Would having to use the test for the rest of your life be a real burden?<br>3. Do you need help to collect urine samples?<br>4. How easy is it to collect urine samples?<br>5. Would you be able to use a smart phone or tablet to send test results through to your GP or clinical?<br>6. If no to question (5), why not?

Analysis

Kalman Filter

A Kalman filter was used, which corresponded to the dynamic linear model (DLM), to obtain conditional expectations of the elements of the state vector. The Kalman filter is an algorithm for recursive calculation of the conditional expectations of the elements of the state vector in a dynamic linear model, at time t, given the observations of the data up to and including time t.

Let $\underline{B}_t$ be the state vector on day t. Let $\underline{b}_{t|s}$ represent the conditional mean and $S_{t|s}$ represent the conditional variance-covariance matrix of $\underline{B}_t$ given the data up to and including time s. Then, after observing the data up to and including day t-1, we have $\underline{b}_{t-1|t-1}$ and $S_{t-1|t-1}$. From these we can compute $\underline{b}_{t|t-1}$ and $S_{t|t-1}$ in a "prediction step". Then, if Y_t is observed, we can compute $\underline{b}_{t|t}$ and $S_{t|t}$ in an "update step". These computations use standard Kalman filter formulae. The new state expectation lies between the predicted local mean $b_{0t|t-1} + b_{1t|t-1}$ and the new observation Y_t and is a better prediction of the current state of the patient than either alone. If no observation is made on day t then we simply have $\underline{b}_{t|t}=\underline{b}_{t|t-1}$ and $S_{t|t}=S_{t|t-1}$ so days when data are missing are handled very easily.

The values which are passed as inputs to the ANN algorithm are the successive values of $b_{1t|t}$, the second element of $\underline{b}_{t|t}$.

Because of the way in which the DLM is constructed, the Kalman filter separates the drift in the local mean, represented by B_{1t}, with expectation $b_{1t|t}$, from the effects of batch changes,

represented by changes in B_{0t}, and from random fluctuations and observational errors, represented by B_{2t} and e_t.

The dynamic linear model (DLM)

A separate univariate DLM is used for each biomarker. The structure is the same in each case but the values of parameters are different for each biomarker. These values are chosen at the offline-learning, or model-fitting, stage. The structure is based on a first-order DLM (West, M. and P. J. Harrison [1997]. Bayesian Forecasting and Dynamic Models [2nd ed.]. New York: Springer-Verlag). This is also known as a ``steady model'' or ``random walk plus noise model''. However, this basic model is modified in three ways. To allow for the effects of batch-changes of the urine tests, one of the variance parameters changes when there is a batch change. Secondly, to separate the ``baseline'' value from the time-varying drift, where it is the latter which is of interest and which is used in the ANN, the baseline and random-walk terms are separated in the state vector. Thirdly, to create a better model of the variation over time, and therefore enable the Kalman filter to separate the changes of interest more effectively, a third term is added to the state vector, representing relatively short-term variation using a stationary first-order autoregressive process.

The state vector $\underline{B}_t$, at time t, has three elements. So $\underline{B}_t=(B_{0t}, B_{1t}, B_{2t})'$. The time steps are days.

The first element, B_{0t}, represents a baseline which is specific to the particular combination of biomarker, patient and test batch. Normally it remains constant so $B_{0t}= B_{0t-1}$. However, when there is a batch change, the baseline is allowed to change, randomly. If there is a batch

change at time t, then $B_{0t}= B_{0t-1}+U_{0t}$, where U_{0t} is a normally distributed random variable with mean 0 and variance v_{0s}. That is, $U_{0t} \sim N(0, v_{0s})$.

The second element, B_{1t}, represents the gradual drift of the local mean. It follows a random walk, so $B_{1t}= B_{1t-1}+U_{1t}$, where $U_{1t} \sim N(0, v_1)$. It is the behaviour of this element which is of particular interest for detecting exacerbations. Note that the local mean of the observations Y_t is $B_{0t}+B_{1t}$, where the observations Y_t are the logarithms of the biomarker concentration measurements.

The third element, B_{2t}, is included to represent fluctuations which are short-term but last longer than a day. We use a stationary first-order autoregressive process. So $B_{2t}= kB_{2t-1}+U_{2t}$, where $U_{2t} \sim N(0, v_2)$. The autoregressive parameter k has modulus less than 1 to give stationarity, so that these fluctuations tend to return to zero and are not confused with the long-term drift B_{1t}. In practice we would choose $0<k<1$ so that the autocorrelation is positive. We can write $v_2=f_b v_1$ where f_b is a "smoothing factor". Larger values of f_b tend to produce smoother series of expected values of B_{1t} from the Kalman filter.

The random changes U_{jt} are mutually independent. That is U_{jt} is independent of U_{hs} unless h=j and s=t. When a new patient series starts, B_{01} is given an initial mean m and an initial variance v_{00} and B_{11} is given an initial mean 0 and an initial variance v_{10}. The stationary distribution of the autoregressive process for B_{2t} has mean 0 and variance $v_2/(1-k^2)$ and this is used for B_{21}.

Finally, the observations depend on the system vector, with $Y_t=B_{0t}+B_{1t}+B_{2t}+e_t$, where e_t is a random error, $e_t \sim N(0, v_e)$ and e_t is independent of e_s unless s=t and of U_{js} for all j and s.

Figure S2. Consort diagrams of discovery phase (a) and validation phase (b)

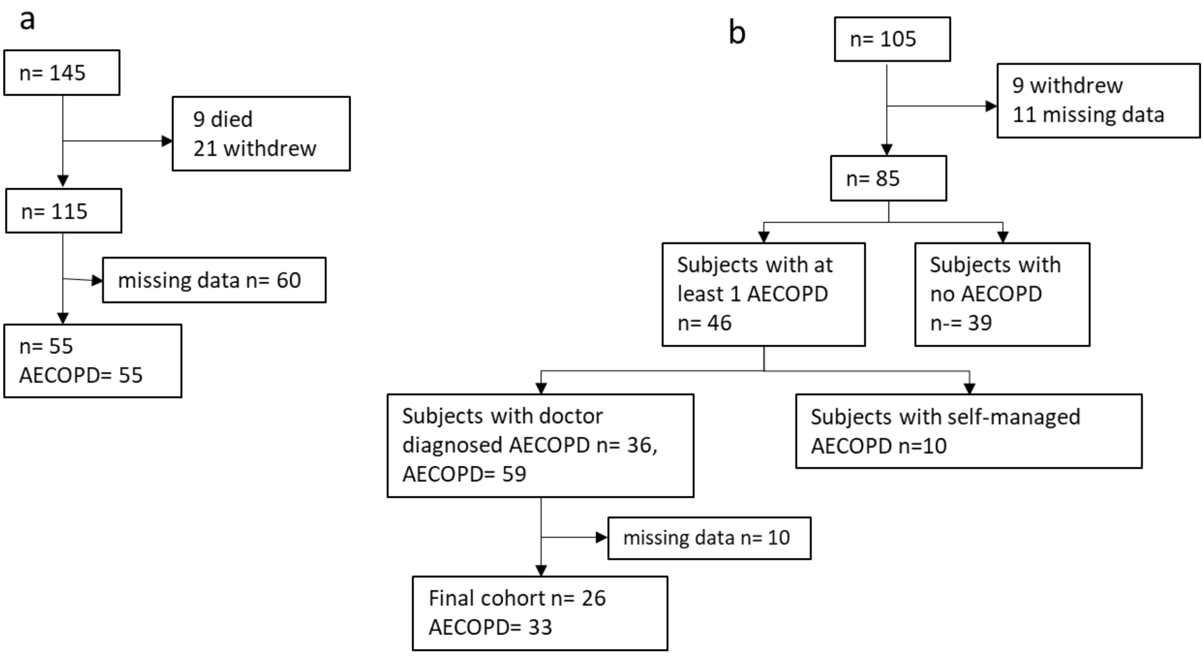

Table S3 Ten candidate urine biomarkers of COPD exacerbation short-listed from a list of 35 biomarkers

Biomarker (ng/mL)	Geometric mean (SD), stable (n=55)	Geometric mean (SD), exacerbation (n=55)	P value	Sensitivity (%)	Specificity (%)	AUC (95% CI)
Fibrinogen	9.54 (4.10)	16.94 (3.90)	<0.01	69.0	50.9	0.64 [0.53 to 0.74]
fmLP	2.93 (2.49)	4.27 (2.90)	<0.01	70.9	49.1	0.61 [0.51 to 0.72]
TIMP2	1.60 (0.00)	2.23 (0.01)	0.028	74.0	40.0	0.64 [0.54 to 0.74]
CRP	0.03 (0.01)	0.13 (0.01)	<0.01	80.0	40.0	0.64 [0.54 to 0.75]
B2M	15.83 (4.86)	42.01 (5.78)	0.013	70.9	65.5	0.63 [0.52 to 0.73]
NGAL	13.13 (2.80)	18.71 (3.54)	<0.01	67.3	65.5	0.65 [0.54 to 0.75]
A1AT	43.16 (4.47)	100.00 (4.08)	<0.01	76.4	58.2	0.72 [0.63 to 0.82]
CC16	14.04 (7.46)	23.35 (5.02)	<0.01	72.7	40.0	0.74 [0.65 to 0.83]
TIMP1	1.00 (0.01)	1.50 (0.01)	<0.01	70.9	50.9	0.62 [0.51 to 0.72]
RBP4	39.18 (0.01)	60.19 (0.01)	<0.01	65.5	56.4	0.68 [0.58 to 0.78]

fMLP, Formyl-Methionine-Leucine-Phenylalanine; CC16, *Club Cell* Protein *16*; TIMP1, Tissue Inhibitor of Metalloproteinase 1; TIMP2, Tissue Inhibitor of Metalloproteinase 2; CRP, C-reactive protein; A1AT, Alpha-a antitrypsin; B2M, *Beta-2-Microglobulin*; NGAL, *Neutrophil gelatinase-associated lipocalin; RBP4,* Retinol Binding Protein 4

Headstart® performance characteristics

Table S4. Assay ranges and lower limits of detection (LLOD)

	ELISA		Multiplex lateral flow	
	**Assay range (multiplied by dilution factor)**	**Lower Limit of detection (LLOD)**	**Assay range (neat urine)**	**Lower Limit of detection (LLOD)**
**A1AT**	7-1600ng/ml	0.164ng/ml	4.1-1000ng/ml	0.59ng/ml
**TIMP-2**	0.6-20ng/ml	1.561ng/ml	0.41-100ng/ml	0.27ng/ml
**NGAL**	8-500ng/ml	0.021ng/ml	2.56-250ng/ml	2.54ng/ml
**Fibrinogen**	1-80ng/ml	0.500ng/ml	10.2-1000ng/ml	5.04ng/ml
**CRP**	0.2-10ng/ml	7.8133ng/ml	0.1-25ng/ml	0.07ng/ml
**RBP4**	47-3000ng/ml	11.720ng/ml	4.1-1000ng/ml	1.87ng/ml
**CC16**	0.3-20ng/ml	0.010ng/ml	10.2-1000ng/ml	9.84ng/ml
**B2M**	1-5000ng/ml	0.006ng/ml	8.2-2000ng/ml	3.44ng/ml
**TIMP-1**	0.3-20ng/ml	14.305ng/ml	0.8-200ng/ml	0.25ng/ml
**fMLP**	2-100ng/ml	0.075ng/ml	1-100ng/ml	0.73ng/ml

Table S5. Standard curves and accuracy

**A1AT r^2 = 0.9988**							
Concentration	1000	400	160	64	25.6	10.24	4.096
Cube units	172.8	1668	141.7	110.6	78.6	47.0	23.3
SD	7.7	7.5	10.4	9.4	6.8	5.4	2.9
%CV	4.5	4.5	7.3	8.5	8.7	11.4	12.4
% accuracy	80.7	126.3	97.2	95.1	102.6	102.4	96.9
**TIMP2 r^2 = 1**							
Concentration	100	40	16	6.4	2.56	1.024	0.41
Cube units	183.9	154.9	114.8	73.8	43.2	26.9	19.8
SD	7.9	5.4	9.3	3.7	3.8	3.0	1.4
%CV	4.3	3.5	8.1	5.1	8.9	11.1	7.0
% accuracy	100.9	98.9	100.3	101.2	98.1	97.5	107.2
**NGAL r^2 = 0.9999**							
Concentration	250	100	40	16	6.4	2.56	
Cube units	163.3	128.3	89.9	55.6	33.8	23.6	
SD	7.0	4.5	6.5	4.0	2.5	3.3	
%CV	4.3	3.5	7.2	7.3	7.5	14.0	
% accuracy	100.6	98.7	101.8	99.2	96.8	107.7	
**Fibrinogen r^2 = 0.9998**							
Concentration	1000	400	160	64	25.6	10.24	
Cube units	151.6	128.9	91.2	56.2	34.9	22.3	
SD	11.1	7.5	11.6	5.1	4.5	3.4	
%CV	7.3	5.8	12.7	9.1	12.8	15.3	
% accuracy	97.99	102.6	98.4	99.1	106.7	91.8	
**CRP r^2 = 0.9997**							
Concentration	25	10	4	1.6	0.64	0.256	0.102
Cube units	242.5	202.2	152.7	92.0	50.4	26.6	15.3
SD	5.8	8.5	9.5	6.0	3.4	3.7	2.1
%CV	2.4	4.2	6.2	6.5	6.7	14.0	13.4
% accuracy	103.2	95.4	103.9	98.2	99.4	99.8	103.7
**RBP4 r^2 = 1**							
Concentration	1000	400	160	64	25.6	10.24	4.096

Cube units	5.3	8.0	18.7	44.1	85.0	113.5	125.0
SD	2.4	11	1.9	5.4	8.0	5.2	12.6
%CV	N/A	14.0	10.3	12.2	9.5	4.6	10.1
% accuracy	102.8	104.2	97.1	101.1	99.4	100.6	99.8
**CC16 r^2 = 0.9989**							
Concentration	1000	400	160	64	25.6	10.24	
Cube units	60.2	94.2	138.0	179.6	212.9	225.5	
SD	7.5	11.6	12.0	14.4	11.3	11.9	
%CV	12.5	12.4	8.7	8.0	5.3	5.3	
% accuracy	1038.5	96.3	100.6	106.4	87.1	80.2	
**B2M r^2 = 1**							
Concentration	2000	800	320	128	51.2	20.48	8.192
Cube units	14.6	25.3	46.1	77.7	116.0	148.8	169.3
SD	1.6	1.8	3.7	6.4	7.1	4.1	13.0
%CV	11.1	7.0	8.1	8.3	6.2	2.7	7.7
% accuracy	100.8	100.2	99.0	100.6	100.1	99.1	100.8
**TIMP1 r^2 = 0.9998**							
Concentration	200	80	32	12·8	5.12	2.048	0.8192
Cube units	247.4	222.2	186.2	131.7	84.0	42.4	18.4
SD	5.9	13.3	6.9	7.4	4.7	4.4	3.2
%CV	2.4	6.0	3.7	5.6	5.6	10.4	17.3
% accuracy	101.3	97.1	103.6	96.7	102.9	98.1	100.7
**fMLP r^2 = 0.9997**							
Concentration	100	40	16	6.4	2.56	1.024	0.4096
Cube units	27.3	36.0	52.7	81.8	110.8	133.6	141.8
SD	1.7	3.8	3.4	6.1	6.9	3.6	8.3
%CV	6.4	10.5	6.5	7.5	6.2	2.7	5.8
% accuracy	104.8	97.0	101.0	98.8	103.3	92.3	108.8

Table S6. Intra-batch reproducibility

63 test devices were sampled from beginning, middle and end from one batch. Samples were tested ranging from low to high concentrations for each analyte (7 standards 9 replicates). %CV was calculated for each individual standard and subsequently an average was calculated from all 7 standards to provide an overall variance. For all 10 biomarkers the average %CV ranged from 6-11% with a min/max ranging from 2%-18%.

**Biomarker**	**average %CV**	**Min %CV**	**Max %CV**
**A1AT**	8.2	4.5	12.4
**TIMP2**	6.9	3.5	11.1
**NGAL**	8.4	3.5	15.1
**FIB**	11.1	5.8	15.3
**CRP**	7.6	2.4	14.0
**T1**	7.3	2.4	17.3
**fMLP**	6.2	2.7	10.5
**RBP4**	9.5	4.6	14.0
**CC16**	7.4	4.4	12.4
**B2M**	6.3	2.7	8.3

Table S7. Inter-batch reproducibility

63 test devices were sampled from beginning, middle and end from three batches. Samples were tested ranging from low to high concentrations for each analyte (7 standards 9 replicates).

%CV was calculated for each individual standard across 3 batches and subsequently an average was calculated from all 7 standards to provide an overall variance. For all 10 biomarkers the average %CV ranged from 10-22% with a min/max ranging from 5%-36%. RBP4 assay has contributed to the high %CV, the remaining 9 assays range from 5%-25%.

Biomarker	average %CV	Min %CV	Max %CV
A1AT	12.9	6.3	23.5
TIMP2	13.5	6.7	24.2
NGAL	12.5	7.0	19.8
FIB	13.2	7.5	21.6
CRP	15.9	5.9	24.6
T1	14.5	6.3	22.4
fMLP	17.4	8.9	23.8
RBP4	21.5	10.8	35.5
CC16	11.5	8.3	14.9
B2M	10.1	5.1	18.7

Figure S3. Headstart® patient acceptability/usability

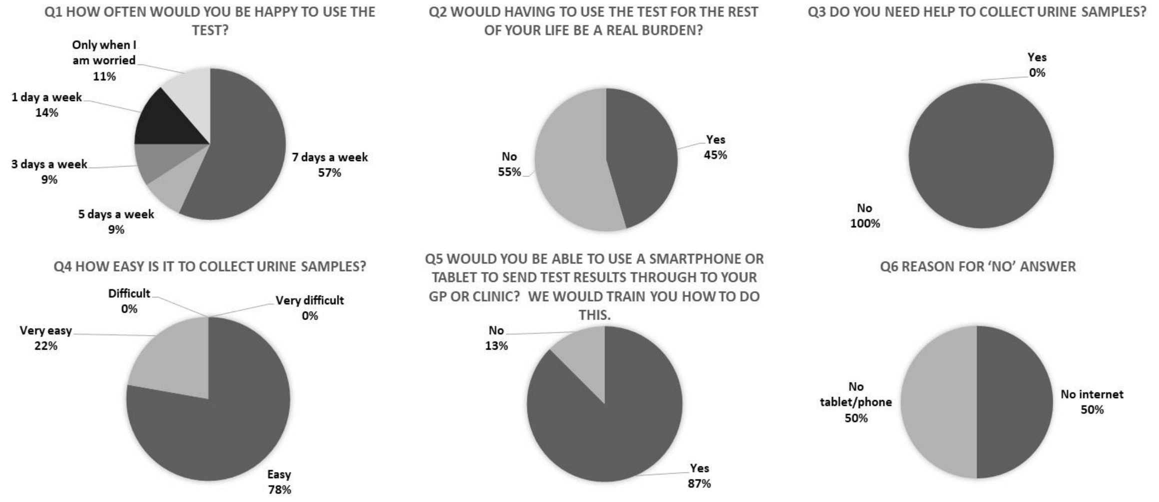

In brief, 45 out of 105 patients provided feedback as shown (Figure S3). When asked how often they would be happy to test, 57% would be happy to perform daily testing; 9% selected 5 or 3 days a week, 14% said they preferred once a week; and 11% said they would take the test only when they were worried. The majority (55%) of patients indicated that having to take the Headstart® test for the rest of their life would not be a burden. To note, the subjects had to complete 2 tests per day, complete a 14-question symptom diary and send an urine sample in the post back to the laboratory, which is a more intense process than what would be required in the final product (1 device only). None of the participants required any help to collect the urine samples; 22% found it very easy to collect the sample and 78% found it easy to collect the sample. With regard to the connectivity, 87% of the patients said they would be able to use a smart phone. Of the 13% who were not keen, 50% had no tablet/smartphone and 50% had no access to the internet.

Figure S4. Headstart® patient compliance

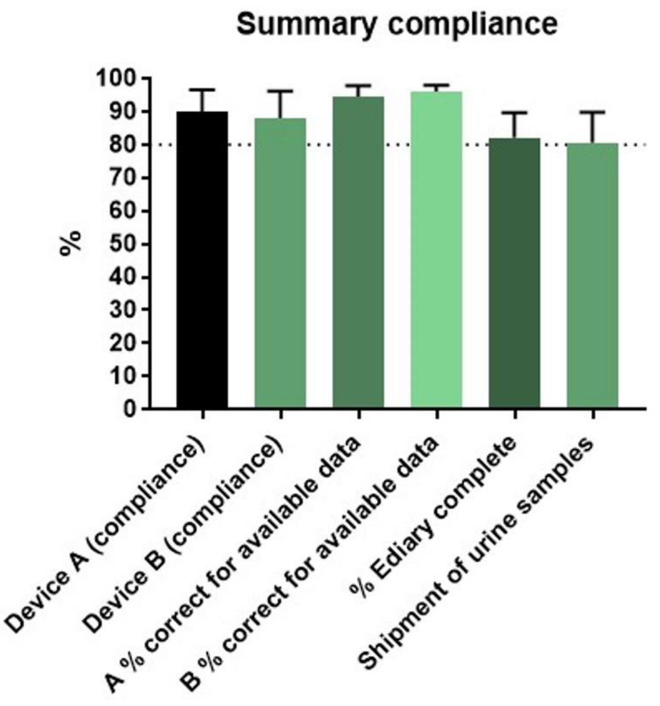

Figure S5. Accuracy measures a) AUC, b) Sensitivity c) Specificity, in the training, validation and test sets for varying exacerbation windows showing optimal window 10-13 days.

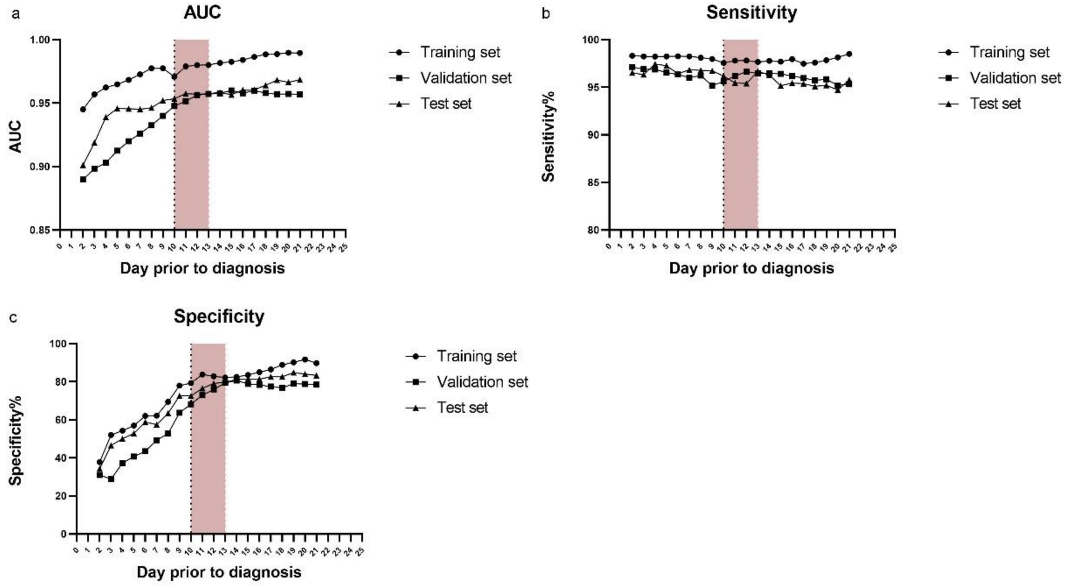

Supplement: Supplementary file 1 [file 00797-2024.SUPPLEMENT.pdf]
